# Activation of basal forebrain cholinergic neurons promotes the attribution of incentive salience to reward cues in male rats

**DOI:** 10.64898/2026.10.20.760494

**Published:** 2026-10-22

**Authors:** Elizabeth A. Rakowski, Christopher P. King, Ashmita Mukherjee, Esther Ruhl, Luke Hannan, Gregory C. Loney, Paul J. Meyer

## Abstract

**Background:**

Reward-associated environmental stimuli (“cues”) can gain incentive salience in some individuals, thereby becoming attractive, reinforcing, and instigators of motivational states. In rodents, the tendency to attribute incentive salience can be measured using a Pavlovian Conditioned Approach (PavCA) procedure, in which approach to a food cue (‘sign-tracking’), relative to approach to the food delivery location (‘goal-tracking’) is the operational definition of the incentive salience attributed to the cue. Growing evidence suggests that acetylcholine promotes this process, thus the current study tested the effect of activating cholinergic neurons in the basal forebrain on sign- and goal-tracking during PavCA.

**Methods:**

We injected a Cre-dependent virus with excitatory Designer Receptors Exclusively Activated by Designer Drugs (DREADDs) into the horizontal limb of the diagonal band of Broca of transgenic rats expressing cre-recombinase in acetylcholine containing neurons. We then injected clozapine-n-oxide (CNO) before each of eight sessions of PavCA. On the ninth session, no CNO was given to test if any effects were immediately reversible.

**Results:**

We found that activation of basal forebrain cholinergic neurons increased sign-tracking over time without an overall effect on goal-tracking, relative to a control group injected with a control virus. Changes in responding from the final CNO session to the saline test did not differ between virus groups.

**Conclusions:**

This effect demonstrates that acetylcholine promotes the attribution of incentive salience to a reward-related cue. Future studies should investigate the effect of inhibiting this area on sign-tracking as well as how activation of this area influences reactivity to cues associated with other rewards such as drugs of abuse.

## Full Text Availability

The license terms selected by the author(s) for this preprint version do not permit archiving in PMC. The full text is available from the preprint server.

